# Curcumin Suppresses Metastasis via Sp-1, FAK Inhibition, and E-Cadherin Upregulation in Colorectal Cancer

**DOI:** 10.1155/2013/541695

**Published:** 2013-07-21

**Authors:** Chun-Chieh Chen, Munisamy Sureshbabul, Huei-Wen Chen, Yu-Shuang Lin, Jen-Yi Lee, Qi-Sheng Hong, Ya-Chien Yang, Sung-Liang Yu

**Affiliations:** ^1^Department of Clinical Laboratory Sciences and Medical Biotechnology, College of Medicine, National Taiwan University, Taipei 100, Taiwan; ^2^Center of Genomic Medicine, National Taiwan University, Taipei 100, Taiwan; ^3^Graduate Institute of Toxicology, College of Medicine, National Taiwan University, Taipei 100, Taiwan; ^4^Department of Laboratory Medicine, National Taiwan University Hospital, Taipei 100, Taiwan; ^5^Graduate Institute of Pathology, College of Medicine, National Taiwan University, Taipei 100, Taiwan; ^6^Center for Optoelectronic Biomedicine, College of Medicine, National Taiwan University, Taipei 100, Taiwan

## Abstract

Colorectal cancer (CRC) is a serious public health problem that results due to changes of diet and various environmental stress factors in the world. Curcumin is a traditional medicine used for treatment of a wide variety of tumors. However, antimetastasis mechanism of curcumin on CRC has not yet been completely investigated. Here, we explored the underlying molecular mechanisms of curcumin on metastasis of CRC cells *in vitro* and *in vivo*. Curcumin significantly inhibits cell migration, invasion, and colony formation *in vitro* and reduces tumor growth and liver metastasis *in vivo*. We found that curcumin suppresses Sp-1 transcriptional activity and Sp-1 regulated genes including ADEM10, calmodulin, EPHB2, HDAC4, and SEPP1 in CRC cells. Curcumin inhibits focal adhesion kinase (FAK) phosphorylation and enhances the expressions of several extracellular matrix components which play a critical role in invasion and metastasis. Curcumin reduces CD24 expression in a dose-dependent manner in CRC cells. Moreover, E-cadherin expression is upregulated by curcumin and serves as an inhibitor of EMT. These results suggest that curcumin executes its antimetastasis function through downregulation of Sp-1, FAK, and CD24 and by promoting E-cadherin expression in CRC cells.

## 1. Introduction

Globally most of the cancers associated with mortality and morbidity arise from metastatic spread of primary tumors. Colorectal cancer (CRC) is the third most prevalent malignancy in human after breast and lung cancers, and statistics suggest that six million people are suffering from CRC and more than 63,900 deaths are expected every year. In 2012, 143,900 people were diagnosed with CRC, and death rate is expected to be 51.690 from CRC in the United States [1]. Recent data suggests that mortality rate from CRC is increasing in Asian countries, particularly in Taiwan, where more than 10,000 patients were diagnosed to be suffering from CRC [2, 3]. CRC affects liver, lung, colon, and rectum, in aged people due to change of their life style and dietary habits. However, the etiology of CRC has not yet been understood clearly. Alternation of normal cells into tumor cells requires several biochemical and phenotype changes in tissue and then spread of individual cell or a small cluster of cancer cells from primary site into distant organs. Mechanism of metastasis is a critical process due to migration of tumor cells from original site into surrounding tissue. Metastasis is classified into at least five different steps: (i) invasion and migration, (ii) intravasation into lymphatic system, (iii) circulation, (iv) extravasation, and (v) colonization. All these steps are mediated by multiple factors such as: growth factors, proteolysis degradation, cell-cell adhesion, cytoskeleton remodeling, and expression of genes which are known to be ultimately involved in metastasis of tumor cells [4]. Transcription factors and their downstream genes involve the modulation of signal transduction pathways leading to either upregulation or downregulation of specific genes expression involved in tumor progression [5–7]. Noteworthy, the focal adhesive kinase (FAK) plays an important role in cytoskeleton remodeling and in regulating the tumor cell invasiveness and metastasis [8, 9]. Moreover, interactions between the cell surface molecule and metalloproteinases (MMPs) require tumor cell invasion and angiogenesis [10]. Downregulation of cell adhesive molecules leads to disruption of adherent junctions resulting in change of epithelial cell morphology, and modulation of phenotype changes epithelial to mesenchymal transition (EMT) [11]. Several factors such as transcription factors, focal adhesion molecule, and cell surface receptors are associated with tumor metastasis. These factors either directly or indirectly participate in the up- or downregulation of specific genes expression in tumor metastasis. Transcription factors associated with housekeeping genes expression are known to be involved in tumor angiogenesis and cell differentiation and to mediate focal adhesive molecule that plays prime role in tumor cell migration and invasion.

Sp-1, a transcription factor that is expressed highly in breast cancer, gastric and thyroid carcinoma cells compared to normal cells, interacts with coactivators and corepressors and thereby activates multiple biological functions such as: cell cycle and tumorigenesis. It also mediates interaction with nuclear factors, protein-protein interaction, and sequence-specific DNA binding [12]. Sp-1 is associated with housekeeping genes expression including vascular epithelial growth factors (VEGF), urokinase plasminogen activator (uPA), urokinase plasminogen activator receptor (uPAR), and epithelial growth factor receptor (EGFR) which are mediated in tumor angiogenesis and cell differentiation [5–7]. Sp-1 also serves as a docking site for binding with CD11b which causes transcriptional activity [13] and is also essential for FAK activation of Krüppel- like factor 8 (KLF8) promoter in human ovarian cancer cells [14]. Acetylation of Sp-1 could modulate Bak and p21 promoter binding activity associated with cell cycle arrest and apoptosis in colon cell lines [15]. These studies implicate that tumor angiogenesis or invasion is associated with Sp-1 activities and its downstream genes expression, and also Sp-1 mediates focal adhesive molecule including FAK which plays prime role in tumor cell migration. It is well known that FAK, a nonreceptor tyrosine kinase protein localized at focal adhesion, serves as a scaffold molecule to mediate several biological functions including cell migration, focal adhesion complex formation. FAK is more highly expressed in breast, ovarian, and CRC metastasis than in normal cells and is activated by its phosphorylation sites thereby interacting with other signals to promote cell migration [16]. It serves as a platform for interaction of ECM and integrins and regulates Src, p130CAS and Grb2, Grb7, and phosphatidylinositol 3-kinase (PI3K) for cell migration and cell cycle progression [9]. It was reported that cell surface molecule such as CD24 mediates c-Src kinase for FAK phosphorylation and paxillin which in turn promote integrin-dependent adhesion and tumor cell invasion and metastasis [17]. This suggests that FAK regulates multiple intracellular signals and cell surface molecule for tumor cell migration. Cell surface molecules regulate the cytoskeleton remodeling and promote cell-matrix adhesion and cell migration, cell invasion, and metastasis. CD24 is one of the major cell surface glycosylated proteins which function as an adhesive molecules of tumor cells [18]. The downregulation of CD24 reduces STAT and FAK activity, decreases cell proliferation, metastasis in human tumor [19], and reduces E-cadherin (E-cad) expression in cultured epithelial cells [20]. These studies suggest that CD24 expression associates with FAK and E-cad function and facilitates the passage of tumor cells in blood stream during metastasis.

It has been reported that loss of cell-cell adhesion is the major process that reduces tumor cell adhesiveness leading to tumor cell growth, invasion, and metastasis [21]. E-cad is a prime component of adherence junction and regulates the tumor cell progression, migration, invasion, and metastasis through maintenance of cell-cell contacts. Noteworthy, the E-cad/*β*-catenin complex stabilizes the cell adherence junctions and also prevents transactivation of gene expression. The *β*-catenin dissociates from E-cad and then translocates into nucleus from cytoplasm, where it binds with LEF1 undergoing transactivation [22]. Downregulation of E-cad could lead to increased *β*-catenin in cytoplasm and is responsible for loss of cell-cell contacts between epithelial cells which in turn change morphology and adhesiveness of EMT [11]. It is well known that epithelial cells are tightly connected with each other by intracellular junctions to avoid cell motility, once epithelial cells lose polarity and intracellular connections and then lead to cell motility and cancer metastasis. Curcumin (CUR) is a hydrophobic polyphenol molecule which persists as a keto-enol tautomerism, and its covalent binding, hydrophobic interactions, and hydrogen bonding play prime role in multiple biological and pharmacological functions [23]. CUR prevents breast, lung, pancreas, and prostate cancer cell proliferation and also exhibits anti-inflammation, antioxidant, antimicrobial, and wound healing activities. CUR interacts with multiple receptors, transcriptional factor, and cytokines and also reduces cell proliferation, angiogenesis, and metastasis [24–28]. Previous studies have implicated that CUR prevents cancer cells migration, invasion, and metastasis through inhibition of PKC, FAK, NF-*κ*B, p65, RhoA, MMP-2, and MMP-7 gene expressions [29]. We have reported that CUR inhibits lung cell invasion and metastasis via upregulation of DnaJ-like heat shock protein 40 (HLJ1) expression and activation of JNK/JunD signaling pathway [27] and also exhibited antitumor caliber in human lung adenocarcinoma cells [28]. Several studies have implicated that CUR mediates the growth factors, cytokine, and transcriptional factors for suppression of tumor apoptosis, cell proliferation, invasion, and metastasis. However, to date there is no direct evidence for CUR effect on transcription factor, focal adhesion molecule, and cell-cell adhesion component for inhibition of tumor metastasis. In this study, we evaluated CUR role on Sp-1 transcription factor, cell adhesion component FAK, CD24 signals, and E-cad expression in CRC cell lines. Inhibition of Sp-1 and promotion of E-cad expression may be an effective strategy for successful treatment of CUR to resistance of CRC metastasis, and CUR is thought to be a potential chemopreventive and therapeutic drug especially in colorectal cancer.

## 2. Materials and Methods

### 2.1. Cell Lines and Cell Culture

The human colon cancer cell lines from NCI-60 panel, including HCT-116, HT-29, HCT-15, HCC-2998, Colo205, Km-12, and SW-620 cells, were maintained in RPMI-1640 medium (Gibco, Grand Island, NY) supplemented with 10% fetal bovine serum ((FBS) Gibco, Grand Island, NY), 1% Antibiotic-Antimycotic (Life Technologies, Carlsbad, CA), 10 mM HEPES (Sigma-Aldrich, St. Louis, MO), and 2 mM L-glutamine (Life Technologies, Carlsbad, CA) at 37°C, in humidified atmosphere of 5% CO_2_.

### 2.2. Cell Viability Assay

CRC cells were seeded at 1 × 10^4^ cells per well in a 96-well plate and incubated overnight. Then, the cells were treated with different concentrations of curcumin (0, 1, 5, 10, 15, 20, 25, 30, and 50 *μ*M) for 24 h. Medium was then removed, and 100 *μ*L of 0.5 mg/mL MTT reagent (Thiazolyl Blue Tetrazolium Bromide; Sigma-Aldrich, St. Louis, MO) was added to each well and reincubated at 37°C incubator for 1.5 h. The MTT reagent was then aspired, and 100 *μ*L DMSO was added to dissolve the Formazan crystal formed by living cells. Absorbance was measured at 570 nm wavelengths by using an ELISA plate reader.

### 2.3. Wound Healing Assay

1 × 10^6^ cells were seeded in each well of 6-well plate and incubate at 37°C incubator for 24 h. The confluent cell monolayers were wounded by a Q-tip. After washing once with PBS, the wells were added with RPMI medium which contained different concentrations of curcumin (0, 5, 10, and 20 *μ*M). Time-lapse images were acquired every 30 min on a Zeiss Axiovert 200 M microscope covered with an incubation system (Carl Zeiss Microimaging, Oberkochen, Germany) for 24 h.

### 2.4. Matrigel Invasion Assay

In invasion assay, transwell membranes (8 *μ*m pore size, 6.5 mm diameter; Corning Costar Corporation, Cambridge, MA) were coated with matrigel (2.5 mg/ml; BD Biosciences Discovery Labware, Bedford, MA), which was diluted in serum-free RPMI medium. 5 × 10^5^ cells seeded onto the matrigel with different concentrations of curcumin (0–20 *μ*M) in serum-free RPMI medium. Lower wells of the transwells contained different concentrations of curcumin in RPMI with 10% FBS. After 18 h incubation, membranes were swabbed with a Q-tip, fixed with methanol, and stained with Giemsa solution. Finally, the invasive cells were counted under microscopy.

### 2.5. Transwell Migration Assay

In migration assay, 3 × 10^5^ cells were seeded onto the top of transwells with different concentrations of curcumin (0–20 M) in serum-free RPMI medium and incubated for 12 h. Lower wells of the transwells contained different concentrations of curcumin in RPMI with 10% FBS. After 12 h incubation, membranes were swabbed with a Q-tip, fixed with methanol, and stained with Giemsa solution. Cells migrated to the lower surface were counted under microscopy.

### 2.6. Anchorage-Independent Colony Formation Assay

For anchorage-independent colony formation assay, six-well plates were first layered with 1 mL 0.7% low-melting point agarose in PBS. In the second layer, 500 HCT-116 cells pre-treated with different concentrations of curcumin (0, 5, 10, and 20 *μ*M) for 24 h were suspended in 1 ml RPMI containing 0.35% low-melting point agarose. 1 ml RPMI medium was covered on the second layer. After 16 days of growth, the cells were washed in PBS, fixed in 4% paraformaldehyde, and stained with 0.1% crystal violet. Finally, the staining solutions were disposed, PBS was added, colonies were counted by naked eye.

### 2.7. *In Vivo* Mouse Model

For *in vivo* metastasis assay, six-week-old severe combined immunodeficiency (SCID) mice supplied by the animal center in the College of Medicine National Taiwan University (Taipei, Taiwan) are housed in a specific-pathogen-free facility. To generate the mouse models with liver metastases derived from human tumor cells, 1 × 10^6^ HCT-116 cells were suspend in 100 *μ*l PBS and injected into the spleen of mice. After one-week recovery, the mice were randomized into control group and treatment group. Curcumin and vehicle control were administered to mice by gastric intubation at the dose of 1 g/kg once daily for 30 days. Mice were sacrificed, and the primary tumors in spleen were harvested and weighed. The livers were also collected and weighed, and the metastatic nodules on livers were counted. Tissues were formalin fixed, paraffin embedded, and sectioned for routine hematoxylin and eosin staining.

### 2.8. RNA Extraction

RNAs were extracted from HCT-116 cells treated with different concentrations of curcumin (0, 5, 10, and 20 *μ*M) for 24 h. In brief, cells in a 10 cm dish were collected with 1 mL of RNA Bee reagent (Tel Test, Friendswood, TX) by a policeman. The cells in RNA bee were then added with 200 *μ*l of chloroform. After the solution was gently mixed and centrifuged, the aqueous layer was collected, mixed with the equal volume of isopropanol, incubated in the −80°C refrigerator for 30 min, and followed by centrifugation at 14,000 ×rpm, 4°C for 15 min. RNA pallets were washed by 75% ethanol and resolved in 50 *μ*L DEPC distilled water. The RNA concentration was determined by spectrophotometer.

### 2.9. Microarray Analysis

12 *μ*g of mRNAs derived from HCT-116 cells treated with DMSO control and curcumin (20 *μ*M) for 24 h was used for mRNA microarray analysis. The mRNA profiles of curcumin treated and DMSO treated HCT-116 cells were analyzed using Affymetrix Human Genome U133 plus 2.0 GeneChip according to the manufacturer's protocols (Santa Clara, CA, http://www.affymetrix.com/) by the Microarray Core Facility of National Research Program for Genomic Medicine of National Science Council in Taiwan. This Affymetrix GeneChip contains 54,675 probe sets to analyze the expression levels of 47,400 transcripts and variants, including 38,500 well-characterized human genes. GeneChips from the hybridization experiments were read by the Affymetrix GeneChip scanner 3000 7G, and raw data were processed using GC-RMA algorithm. The raw data were also analyzed by GeneSpring GX software (Silicon Genetics, Redwood City, CA). The GO biological processes of genes with greater than twofold change in curcumin treated cells compared with DMSO treated cells were analyzed using MetaCore Gene Set Enrichment Analysis (GSEA) software program (GeneGo Inc., St Joseph, MI).

### 2.10. Quantitative Real-Time PCR

To confirm the expression patterns of upregulated or downregulated genes after curcumin treatment, the selected cell adhesion- and metastasis-related genes were chosen for further analysis using quantitative real-time PCR (qRT-PCR) in a 96-well format. Briefly, all reactions were carried out in 20 *μ*L solution containing 10 *μ*L of 2X SYBR Green PCR Master Mix (Applied Biosystems, Foster City, CA). The TATA box binding protein (TBP) was quantified as an internal control using the primers described in a previous report [27]. DNA amplification was carried out using ABI 7900 Sequence Detection System (Applied Biosystems, Foster City, CA), and the detection was carried out by measuring the binding of the fluorescence dye SYBR Green I to double-stranded DNA. An amplification plot of the fluorescence signal versus cycle number was drawn. In the initial cycles of PCR, there was little change in fluorescence signal. This defined the baseline for the amplification plot. An increase in fluorescence above the baseline indicated the detection of accumulated PCR product. A fixed fluorescence threshold was set above the baseline in the exponential phase of the PCR. The parameter CT (threshold cycle) was defined as the fractional cycle number at which the fluorescence passed the fixed thresholds. The differences (ΔCT) between the mean values in triplicate samples of tested gene cDNA and those of TBP were calculated by Microsoft Excel, and the relative quantitation value was defined as 2 − ΔCT × K, where K is a constant.

### 2.11. Western Blot Analysis

Cells cultured in 10 cm dish were treated with different concentrations (0, 5, 10, and 20 *μ*M) of curcumin for 24 h. Cells were then disrupted in 500 *μ*L of lysis buffer (50 mM Tris/HCl, pH 7.4), 1% (v/v) Triton × 100, 10% glycerol, 150 mM NaCl, 1 mM EDTA, 20 *μ*g/mL leupeptin, 1 mM PMSF (phenylmethylsulfonyl fluoride), 20 *μ*g/mL aprotinin, and 20 *μ*g/ml pepstain and centrifuged at 15,000 ×g for 30 min at 4°C. The proteins were separated on a 12% SDS polyacrylamide gel and then transferred onto nitrocellulose membranes with 400 mA/cm^2^, 100 volts for 60 min. The membranes were first blocked with 5% skim milk in TBST (0.2 M NaCl, 10 mM Tris, pH 7.4, and 0.1% Tween 20) for 1 h and sequentially incubated with anti-FAK and anti-*β*-actin monoclonal antibody (Life Technologies, Carlsbad, CA) in 0.5% skim milk for 16 h at 4°C. The membranes were then washed three times with TBST, followed by incubation with horseradish peroxidase-conjugated secondary antibody for 1 h at room temperature. Bound antibody was detected using the Enhanced Chemiluminescence System (Santa Cruz Biotechnologies, Santa Cruz, CA). Chemiluminescent signals were captured using the Fujifilm LAS 3000 system (Fujifilm, Tokyo, Japan).

### 2.12. Cell Adhesion Assay

The 96-well plate (Costar, Cambridge, MA) was coated with fibronectin (25 *μ*g/mL), collagen I (100 *μ*M/mL), collagen III (100 *μ*M/mL), collagen IV (100 *μ*M/mL), collagen IX (100 *μ*M/mL), laminin (25 *μ*g/mL), and negative control (BSA 40 *μ*M/mL) at 4°C overnight. After removing these coating extracellular matrix (ECM), the plate was blocked by 1% BSA at 4°C in CO_2_ incubator for 4 h. HCT-116 cells were pretreated with different concentrations of curcumin (0, 5, 10, and 20 *μ*M) for 24 h and then changed into serum-free medium for 30 min. Cells were trypsinized, and cell numbers were adjusted to 2 × 10^5^ cells/ml in serum-free medium. After removing the blocking buffer, 2 × 10^4^ cells in 100 *μ*l serum-free medium were added in each well of the 96-well plate. The plate was incubated in CO_2_ incubator at 37°C for 60 minutes. Adhesive cells were washed with washing buffer (PBS) 2-3 times, fixed with 4% paraformaldehyde, and stained with Hoechst dye. Images of cells in each well of the 96-well plate were acquired, and the number of adhesive cells was calculated by High Content System (HCS).

### 2.13. Sp-1 Luciferase Assay

5 × 10^5^ 293T cells in 6-well plate were transient transfected with Sp-1 reporter plasmids (2 *μ*g) and Renilla control vectors (0.2 *μ*g). After 24 h, the cells were treated with different concentrations of curcumin (0, 5, 10, and 20 *μ*M) for further 24 h. Then, the activities of firefly and Renilla luciferase were quantified by dual luciferase assay system (Promega, Madison, WI). The transcriptional activity of Sp-1 was expressed as a ratio of firefly : Renilla luciferase activity.

## 3. Results

### 3.1. Curcumin Inhibits Proliferation, Migration, Invasion, and Colony Formation of CRC Cell Lines

**Figure 1:**
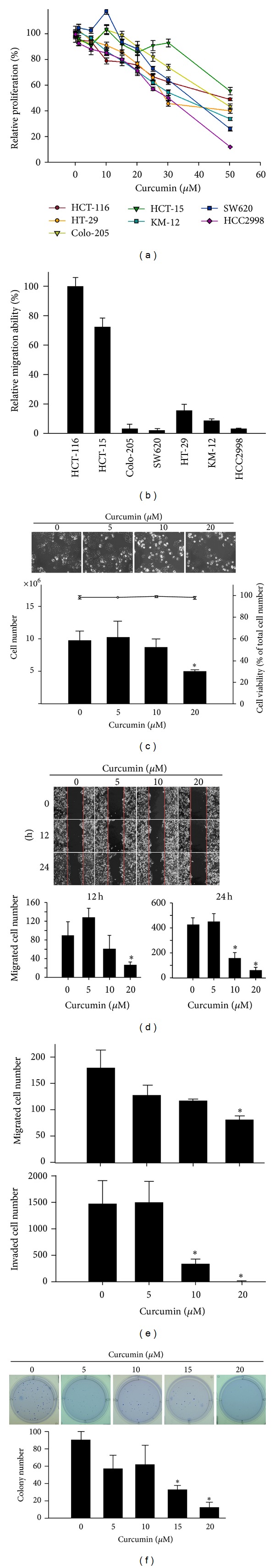
Curcumin inhibits proliferation, migration, invasion, and colony formation of CRC cell lines. (a) Proliferation inhibitory effects of curcumin on CRC cell lines. Proliferation of CRC cells was detected in HCT-116, HT-29, Colo-205, HCT-15, KM-12, SW620, and HCC2998 cell lines treated with different concentrations of curcumin (0–50 *μ*M) for 24 h, and cell viability was determined by MTT assay. Data are presented as mean ± SD (*n* = 6). (b) Cell migration abilities of CRC cell lines. The migration-based cell motility screen was chosen to determine the relative cell motility of the 7 CRC cell lines. 5 × 10^5^ cells in serum-free RPMI medium were seeded onto the top of transwells and incubated for 12 h. Cells migrated to the lower surface were stained with GIMSA stain and counted under microscopy. Migrated cell number was normalized to migrated cells of HCT-116 cell line. Data are presented as mean ± SD (*n* = 3). (c) Effects of curcumin on cell morphology and cell viability of HCT-116 cells. HCT-116 cells seeding in a 6-well plate were treated with different concentrations of curcumin (0, 5, 10, and 20 *μ*M) for 24 h. Images of cell morphology were captured using Zeiss Axiovert 200 M microscope under 100x magnification (Top). After 24 h curcumin treatment, cell number of HCT-116 was measured using trypan blue exclusion and cell counting method (Bottom). Data are presented as mean ± SD (*n* = 3). *P < 0.05. (d) Curcumin inhibits HCT-116 cells migration in wound healing assay. Inhibitory of curcumin on HCT-116 cells, and monolayers were wounded with a sterile 200 *μ*l pipette tip, and the cells were treated with different concentrations of curcumin (0, 5, 10, and 20 *μ*M). Cells were photographed at 0 h and every 30 min to 24 h. Images showed HCT-116 cells at 0, 12, and 24 h under 100x magnification (Top). Number of migrated cells within the wound area was counted at the time point 12 h (bottom left) and 24 h (bottom right). Data are presented as mean ± SD (*n* = 3). *P < 0.05. (e) Curcumin suppresses HCT-116 cells motility in transwell migration and invasion assays. HCT-116 cells with serum-free media containing different concentrations of curcumin (0, 5, 10, and 20 *μ*M) were seeded into the upper chamber of the transwell. Bottom wells were filled with complete media. After 12 h incubation, cells migrated through the transwell membrane were fixed and stained with Giemsa solution. Migrated cell numbers were counted under a light microscope with 200x magnification (Top). After 18 h incubation, the cells invaded through the matrigel membrane were stained and counted (Bottom). Data are presented as mean ± SD (*n* = 3). *P < 0.05. (f) Curcumin inhibits anchorage-independent growth of HCT-116 cells in soft agar assay. 500 HCT-116 cells pretreated with different concentrations of curcumin (0, 5, 10, and 20 *μ*M) for 24 h were suspended in growth medium containing 0.35% low-melting point agarose and seeded in 6-well plates coated with a basal layer of 0.7% low-melting point agarose. Colony assay was set up in triplicate. 2 weeks later, colonies were fixed and stained with crystal violet. Representative soft agar culture dishes showing reduced colony formation dosedependently (top). Graphic presentation of representative results of colony formation in soft agar (bottom). Data are presented as mean ± SD (*n* = 3). *P < 0.05.

The current study was conducted to delineate the underlying molecular mechanism of CUR on CRC metastasis. First, we attempted to observe the biological effect of CUR on CRC cell viability. The CRC cell lines (HCT-116, HT-29, Colo-205, HCT-15, KM-12, SW620, and HCC2998) were exposed to different concentrations of CUR for 24 h, and performed MTT assay (Figure 1(a)). The results suggest that HCT-116 cells proliferation was inhibited by CUR in a dose-dependent manner. The IC_50_ value of CUR was nearly 50 *μ*M in HCT-116 cell for 24 h. We tested the cell migration ability of seven CRC cell lines using transwell migration assay. Among these, HCT-116 cell line has shown the highest migration levels, while HCT-15 and HT-29 cells displayed moderate migration levels (Figure 1(b)). Hence, we chose HCT-116 cell line and four sublethal doses of CUR (0, 5, 10, and 20 *μ*M) for further investigations. The cytotoxic effect of CUR on HCT-116 cells was analyzed by trypan blue exclusion and cell counting method. The result showed no cytotoxic effect of CUR on HCT-116 cells morphology at below 20 *μ*M concentration for 24 h (Figure 1(c), top). The cells number and viability measured by trypan blue exclusion showed no significant change below 10 *μ*M CUR treatment but decreased to nearly 50% of control in the dose of 20 *μ*M (Figure 1(c), bottom). These data suggest that 20 *μ*M CUR had no toxicity in HCT-116 cells, and the inhibition of cell proliferation was nearly 50%. It is well known that metastasis is mostly dependent on tumor cell migration and invasion; therefore we are interested to observe the CUR role on HCT-116 cell migration using wound healing assay. Cells migrated into the wound area were visualized under Carl-Zeiss inverted microscope at different time points (Figure 1(d), top). The result suggests that HCT-116 cell migration was significantly inhibited by CUR (Figure 1(d), bottom). To further confirm the effects of CUR on cell motility, we used transwell migration (Figure 1(e), top) and invasion (Figure 1(e), bottom) assay as well as zymography to determine whether cell mobility and the activities of MMP2 and MMP9 (see Supplementary Data S1 available online at http://dx.doi.org/10.1155/2013/541695) are influenced by CUR. The data revealed that CUR could dose dependently inhibit the migration (nearly 55% of inhibition at 20 *μ*M) and also remarkably suppress the invasion ability (nearly 89% of inhibition at 10 *μ*M and 99% of inhibition at 20 *μ*M) and the activities of MMP2 and MMP9 of HCT-116 cells (Figure 1(e) and Supplementary Data S1). Tumor metastasis is highly dependent on cancer cell invasion and anchorage-independent tumorigenesis. To investigate whether CUR regulates the anchorage-independent colony growth of tumor cells, different concentrations of CUR (5–20 *μ*M) were used to treat HCT-116 cells for two weeks using soft agar assay, which resulted in reduced colony formation of HCT-116 cells (Figure 1(f)). Taken together, these results suggest that CUR significantly inhibits tumor cell proliferation, migration, and invasion and also suppresses colony formation in HCT-116 cells.

### 3.2. Curcumin Inhibits Tumor Growth and Liver Metastasis *In Vivo*

**Figure 2:**
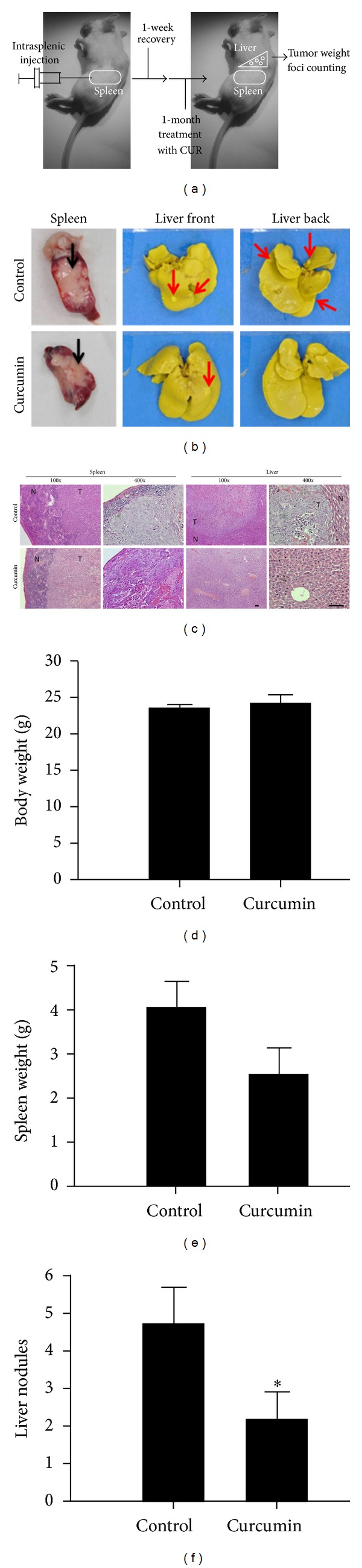
Curcumin inhibits tumor growth and liver metastasis *in vivo*. (a) Illustration of the experimental design showed the metastatic animal model. (b) Red arrow indicates the invaded tumor nodules from spleen to liver. (c) H&E staining of paraffin-embedded tumor sections of vehicle control and curcumin treated mice. Images were acquired under an inverted microscope at 100x and 400x. T: tumor; N: normal tissue (scale bars: 10 *μ*m). (d) The body weight of mice was not affected by CUR treatment. (e) Curcumin inhibits primary tumor growth in spleen (normalized with body weight) and (f) liver metastasis. *P < 0.05.

Further, to explore whether CUR could exhibit antimetastasis effect on CRC *in vivo*, here, the HCT-116 cells were injected into the spleen of severe combined immune-deficient (SCID) mice, then spleen metastases were observed after one week, further mice were treated with vehicle control or CUR (1 g/kg) for one month, finally the mice were sacrificed, and the primary tumors in spleen were harvested and weighed. The schematic illustration of *in vivo* experiment was shown in Figure 2(a), and the metastatic nodules on livers were indicated (Figure 2(b)). The pathologies of primary tumors and metastatic nodules were examined using H&E staining (Figure 2(c)). CUR treatment (*n* = 10) did not influence the body weight of mice suggesting that mice health was not affected by CUR treatment (Figure 2(d)). In this study, CUR significantly reduced primary tumor growth (Figure 2(e)) and number of liver metastatic nodules (Figure 2(f)) compared to control group (*n* = 8). The results clearly demonstrate that CUR inhibits tumor growth and cancer metastasis of CRC *in vivo* model.

### 3.3. Microarray Analysis of Gene Expression of HCT-116 Cells after Treatment with Curcumin

**Figure 3:**
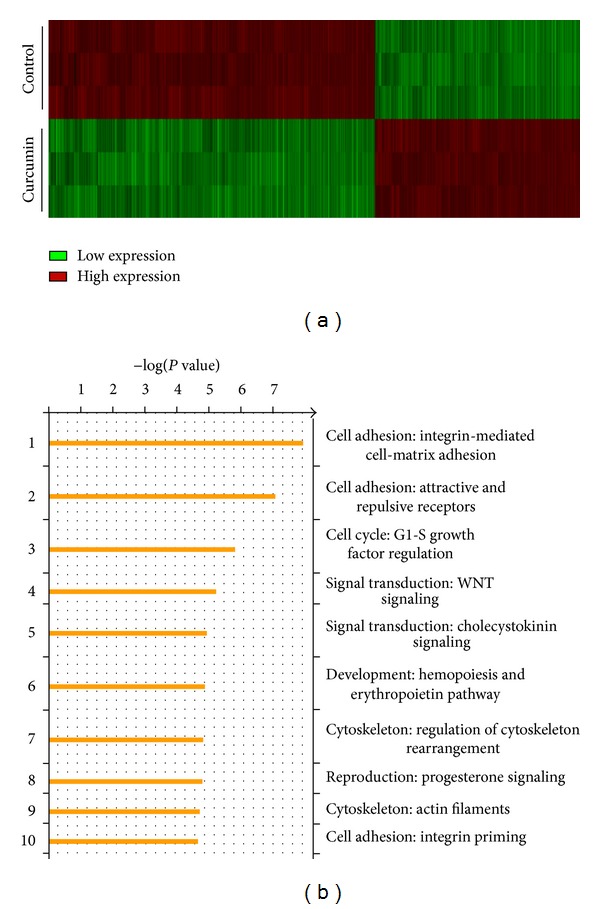
Gene expression profiles and pathway analysis of curcumin-regulated genes in HCT116 cells. (a) Affymetrix microarray was used to screen gene expression of control and curcumin treated HCT-116 cells, and the heatmap of gene expression profile was shown. (b) Curcumin-regulated genes were grouped and done by pathway analysis via MetaCore software, and the pathways involved were ranked.

The antimetastasis of CUR in HCT-116 cells was investigated by Affymetrix Human Genome U133 Plus 2.0 Array Chip. HCT-116 cells were treated with or without CUR (20 *μ*M) for 24 h, and the primary analysis was done by GeneSpring GX program (Figure 3(a)). After RMA normalization and FDR correction, the functional analysis was done by MetaCore to identify the most relevant signaling pathways (Figure 3(b)). We observed 1239 genes that show more than twofold change including transcription factors, Sp-1, in HCT-116 cells in response to CUR (20 *μ*M) (Table 1). These genes were categorized based on their cellular activities including the cell motility, cytoskeleton remodeling, cell adhesion, and chemotaxis signaling pathways. We selected CUR-regulated genes that were involved in tumor cell migration, invasion, and metastasis and further confirmed by qRT-PCR with specific primers (Table 2).

### 3.4. Curcumin Inhibits CD24 Expression in HCT-116 Cells

**Figure 4:**
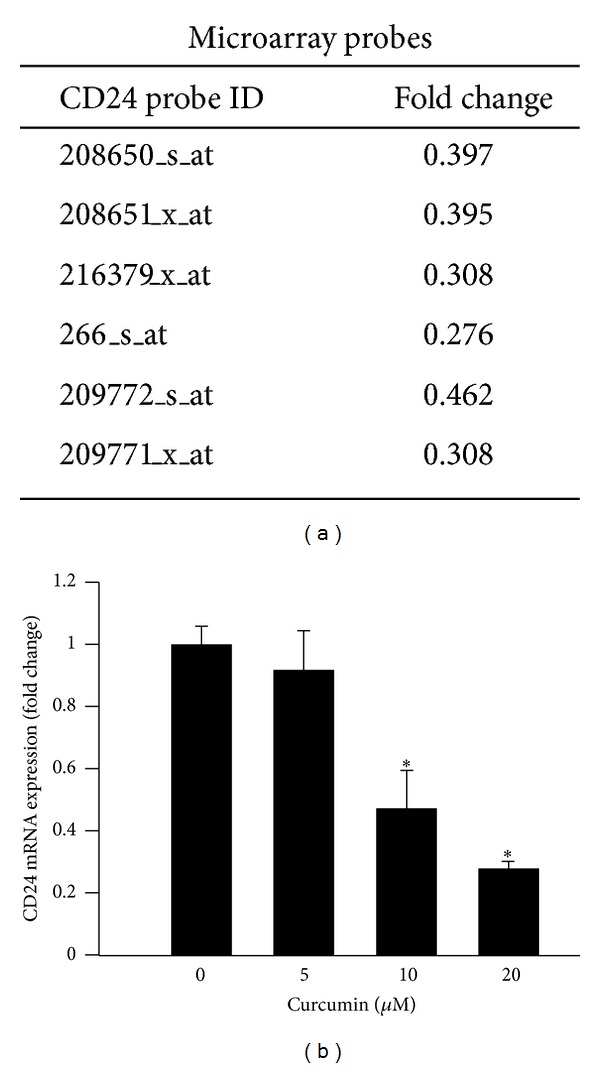
Curcumin Inhibits CD24 expression in HCT-116 cells. (a) Gene expression fold change of CD24 was detected by Affymetrix microarray, indicating that CD24 was downregulated in 6 different probes. (b) Gene expression level of CD24 was validated by qRT-PCR. Curcumin dose dependently inhibited the expression of CD24 mRNA which was consistent with microarray data. Data are presented as mean ± SD (*n* = 3). *P < 0.05.

We investigated whether CUR induces up- or downregulation of gene expression to gain knowledge for choosing the novel gene related to CRC metastasis. Based on the microarray data we observed the following antimetastasis genes such as: AKR1B10, ARHGDIA, CD24, LEMD1, and HDAC4. Among them, we were mostly interested in CD24 which is a cancer cell surface marker associated with CRC invasiveness, differentiation, and tumor metastasis. CD24 expression was detected in microarray analysis, which consistently showed downregulation by six different probes (Figure 4(a)). We next performed qRT-PCR to validate whether CD24 was down-regulated by CUR in HCT-116 cells; results indicated that CD24 expression was significantly reduced by CUR in a dose-dependent manner (Figure 4(b)). This result suggests that CUR inhibited CD24 expression in CRC cells.

### 3.5. Curcumin Suppresses Sp-1 Transcriptional Activity and Its Downstream Gene Expressions

**Figure 5:**
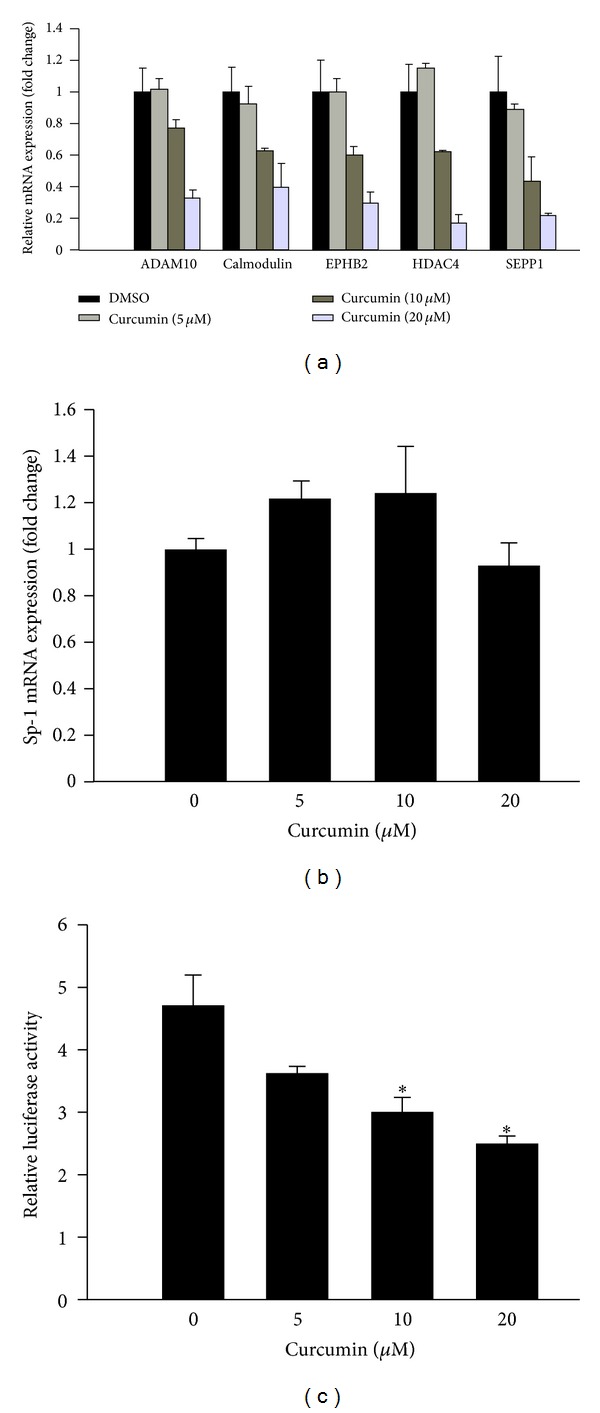
Sp-1 transcriptional regulation via curcumin treatment. (a) Gene expression fold changes of five Sp-1 downstream genes, containing ADAM10, calmodulin, EPHB2, HDAC4, and SEPP1, were determined using qRT-PCR. Five genes transcriptionally activated by Sp-1 showed uniformly suppression via curcumin treatment. (b) The gene expression level of Sp-1 was measured by qRT-PCR. (c) Luciferase reporter assay quantifying the Sp-1 transcriptional activity changes in curcumin treated cells. HEK293T cells transiently transfected with Sp-1 reporter plasmid and Renilla control plasmid were treated with different concentrations of curcumin. The firefly and Renilla luciferase activities of the cell extracts were quantified using the Dual-Glo luciferase assay system. Data are presented as the ratio of firefly:Renilla luciferase activity. *P < 0.05.

Further, we analyzed the underlying molecular mechanism of transcription genes associated with tumor cell migration, invasion, and metastasis. Sp-1 is mostly influenced by transcription factor that activates many genes containing GC box in their promoters involved in tumor progression. Our qRT-PCR results demonstrated that CUR suppresses the Sp-1 activation and its downstream genes including ADEM10, calmodulin, EPHB2, HDAC4, and SEPP1 in a concentration-dependent manner (Figure 5(a)). Cells treated with different concentrations of CUR suggested that expression of Sp-1 was not affected by CUR (Figure 5(b)). To directly clarify the possible role of CUR on Sp-1 activation, the HCT-116 cells were transiently transfected with PGL3-Sp-1 luciferase reporter plasmid which served as an indicator of Sp-1 activation, and then cells were treated with different concentrations of CUR for 24 h. Results suggested that Sp-1 luciferase activity was inhibited by CUR in a concentration-dependent manner (Figure 5(c)). Our results indicate that CUR inhibits Sp-1 transcription factor and its downstream gene expressions which are involved in the metastasis. These data clearly suggest that CUR inhibits CRC metastasis through suppression of Sp-1 and its downstream gene expressions in CRC cell line.

### 3.6. Effect of Curcumin on FAK Regulated Cytoskeleton Remodeling and Cell Adhesion Pathways

**Figure 6:**
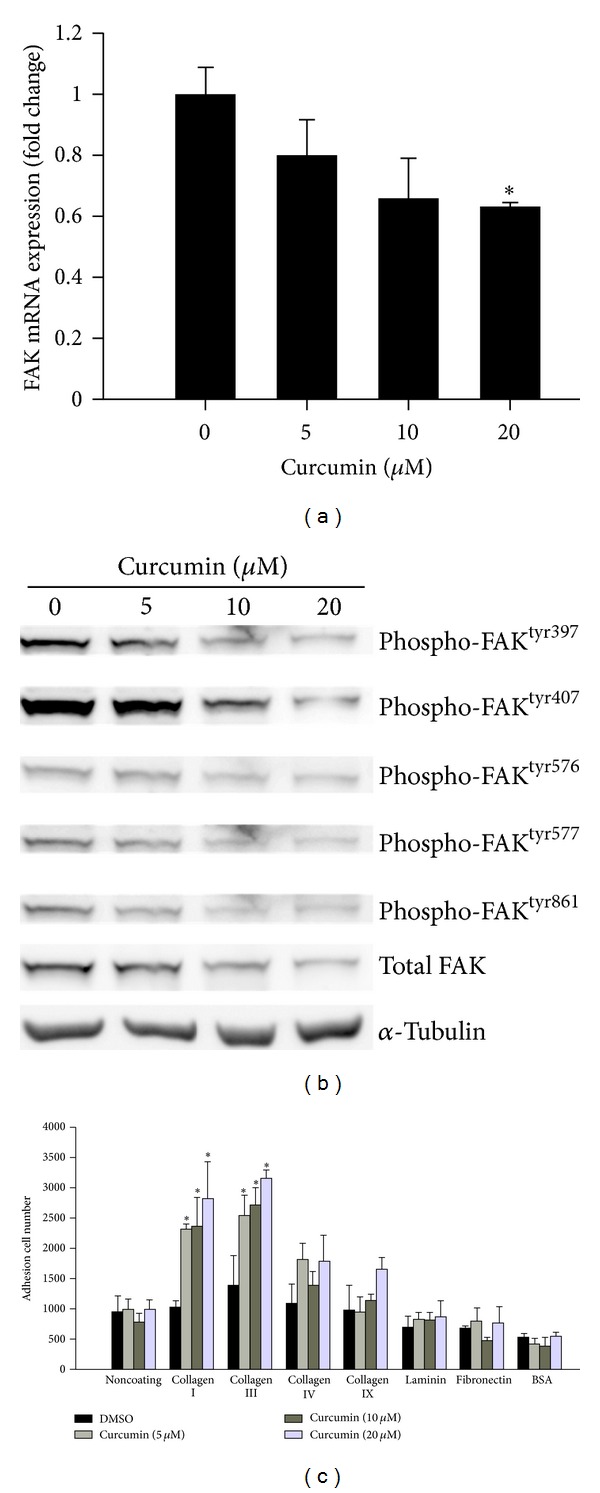
The effect of curcumin on FAK expression and cell adhesion. (a) FAK mRNA levels were suppressed under curcumin treatment and detected by qRT-PCR. (b) Curcumin inhibits the protein expression and phosphorylation of FAK. HCT-116 cells were treated with different concentrations of curcumin for 24 h. The cell lysates were harvested and analyzed by specific antibodies against FAK and different tyrosine phosphorylation sites of FAK, including 397, 407, 576, 577, and 861. (c) Curcumin enhances cell adhesion ability. After pretreatment with serial curcumin for 24 h, 2 × 10^4^ cells were seeded on 96-well plates coated with different ECM, including collagen I, collagen III, collagen IV, collagen IX, laminin, and BSA as negative control. The noncoating control at the first lane was performed to control added cell number. Cells were allowed to adhere for 1 h at 37°C and then washed twice with PBS. Bound cells were stained by DAPI and analyzed by high content system. Data are presented as mean ± SD (*n* = 3). *P < 0.05.

We further analyzed antimetastasis effect of CUR on uncovered genes that contribute in tumor metastasis. MetaCore analysis showed that cytoskeleton remodeling, cell adhesion, and chemotaxis signaling pathways were associated with CUR mediated tumor metastasis (Figure 3(b)). Here, our result showed that CUR significantly suppressed FAK expression in a dose-dependent manner (Figure 6(a)) and also inhibited its phosphorylation (sites on Tyr397, 407, 576, 577, and 861) in HCT-116 cells (Figure 6(b)). FAK promotes cell invasion and adhesion through regulating focal adhesion turnover dynamics and cytoskeleton polymerization; we speculated that downregulation of FAK may be involved in cancer cell adhesion ability. After pretreatment with different concentrations of CUR for 24 h, HCT-116 cells were seeded on 96-well plates coated with different ECM, and then bound cells were stained by DAPI and analyzed by HCS. This result showed the enhancement of cell adhesion ability dose dependently after CUR treatment. We observed that CUR enhances cell adhesion ability through induction of ECM components collagen I, collagen III, collagen IV, collagen IX, laminin, and fibronectin in a concentration-dependent manner (Figure 6(c)). Taken together, these results suggested that CUR suppresses FAK activity via inhibition of its phosphorylation sites and also induces ECM components to enhance cell adhesion ability, thereby preventing detachment of tumor cells and cell migration. Inhibition of FAK expression leads to increased cell adhesion which might be potential mechanism of the antimetastasis effect of CUR.

### 3.7. The Potential Inhibitory Effect of Curcumin on Epithelial-Mesenchymal Transition

**Figure 7:**
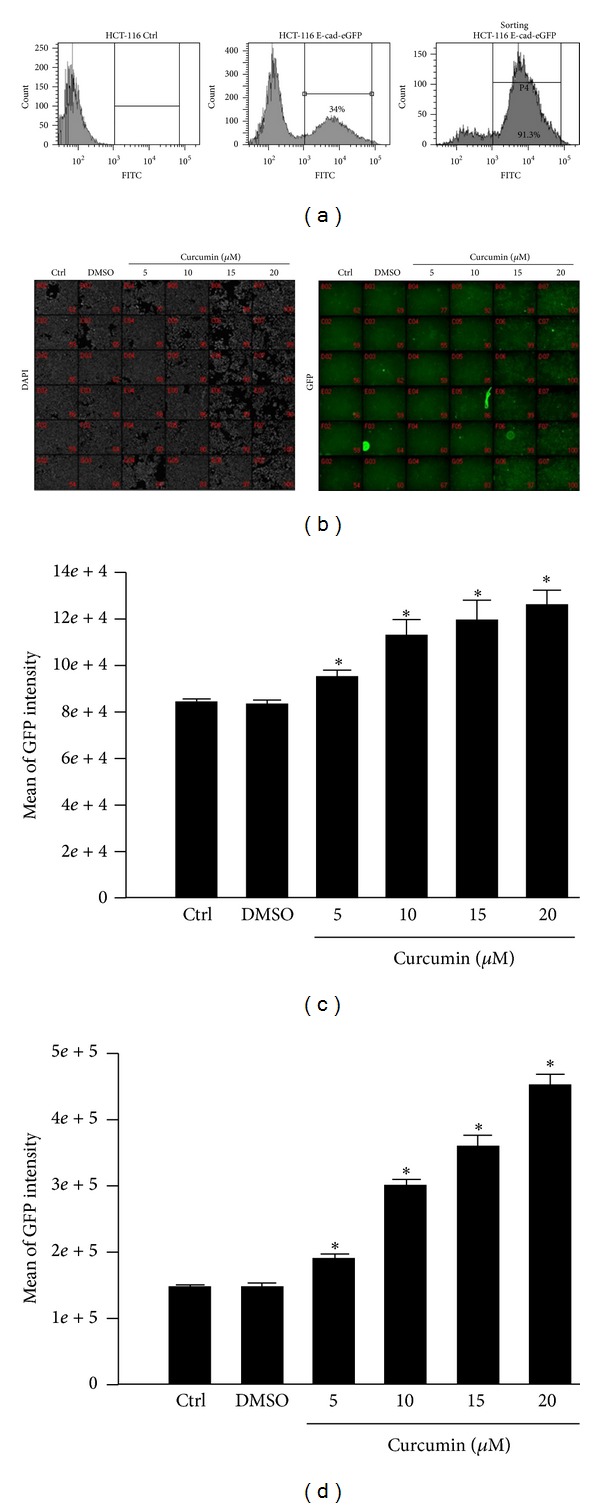
The potential inhibitory effect of curcumin on epithelial-mesenchymal transition. (a) The percentage of GFP positive cells was increased from 34% to 91.3% after fluorescence-activated cell sorting. (b) Curcumin increases GFP intensity of the selected HCT-116 stable cells. 1 × 10^4^ cells were seeded in 96-well plates overnight. After incubation with different doses of curcumin for 24 h, the cells were fixed and stained with DAPI. The images of 96-well plates were acquired, and the fluorescence intensity of each cell was analyzed by HCS. (c) Curcumin increases E-cadherin promoter activity in dose-dependent manner. (d) Curcumin increases thrombomodulin promoter activity in dose-dependent manner. Data are presented as mean ± SD (*n* = 3). *P < 0.05.

EMT is a loss of epithelial cells morphology to acquire mesenchymal-like phenotype and plays a critical role in cancer progression and metastasis. On the other hand, to explore whether EMT was suppressed by CUR in CRC cell lines, we used two EMT markers, E-cad and thrombomodulin promoter, to examine the influence of CUR on EMT process. HCT-116 cells were transfected with E-cad promoter-GFP reporter plasmid or thrombomodulin promoter TM1519-GFP reporter plasmid. Stable cells were selected by using G418 treatment and fluorescence-activated cell sorting (Figure 7(a)). The GFP positive cells were selected for the following CUR treatment. The images of the fluorescence intensity of each cell were analyzed by high content system (HCS) (Figure 7(b)). The results showed that CUR dose dependently increases both of the E-cad promoter (Figure 7(c)) and thrombomodulin promoter activity (Figure 7(d)). These data indicate that CUR prevents EMT through induction of E-cad and thrombomodulin expression in CRC cell line.

## 4. Discussion

Natural therapeutic drugs prevent tumor cell progression, invasion, and metastasis through modulation of growth factors, focal adhesive molecules, cell surface molecules, apoptosis-related genes, transcription factors, and signal transduction pathways. We already have reported that CUR induces apoptosis, causes downregulation of EGFR, Akt, cMET cyclin D1, and PCNA protein expression in CL-5 xenograft tumors [28], and inhibits lung cell invasion and metastasis through upregulation of HLJ1 expression in tumor cells [27]. CUR inhibits cell proliferation, cell cycle arrest and stimulates apoptosis via modulation of wide range of transcription factors, including NF-*κ*B, AP-1, Erg-1, STAT-3, p53, *β*-catenin, Notch-1, Hif-1, and PPAR-*α* [30]. However, the molecular mechanism of CUR on Sp-1, focal adhesion molecules, and cell-cell adhesion component has not been implicated. Very little information is available regarding the effect of CUR on Sp-1 in CRC cells. Several studies have reported that Sp-1 is responsible for upregulation of housekeeping genes (VEGF, uPA, uPAR, and EGFR) which participate in tumor cell angiogenesis and metastasis [5–7]. Hence, inhibition of Sp-1 and its housekeeping gene expressions is an important hypothesis to prevents tumor formation, migration, and invasion [31, 32]. Our microarray, qRT-PCR, and luciferase assay data showed that CUR suppresses the Sp-1 activation and its downstream genes including ADEM10, calmodulin, EPHB2, HDAC4, and SEPP1 in a concentration-dependent manner in CRC cell lines; these results are consistent with other studies where it has been reported that CUR suppresses the Sp-1 activity in bladder cancer [33] and decreases DNA binding activity of Sp-1 in NSCLC cells [34]. Our data raise the possibility that CUR may prevent metastasis ability of CRC cells *in vitro* and *in vivo* through inhibition of Sp-1 and its housekeeping gene expressions which are known to be involved in tumor progression. Moreover, the CUR significantly reduced colony formation; results are in agreement with other studies demonstrating that downregulation of Sp-1 prevents the colony formation in a patient derived fibrocarcinoma cell line [35]. Our results suggest that downregulation of Sp-1 and its downstream genes leads to inhibition of colony formation, and transactivation gene expression might be a possible mechanism for preventing the colorectal tumor cell metastasis, because the Sp-1 serves as a master switch to turn on and off the housekeeping gene expressions which play vital roles in tumorigenesis [35].

### 4.1. Potential Role of FAK Signaling in the Antimetastasis Effect of Curcumin

Studies have implicated that FAK mediates cell proliferation through upregulation of cyclin D3, PKC, and PI3K/Akt signaling pathways [36] and by interacting with Src, Grb, PI3K, and P130CAS which promotes cell migration [9]. Loss of FAK activity is associated with suppression of tumor formation [37], reduced cell attachment with ECM, and reduced cell adhesion to collagen type II [38]. These studies implicated that FAK expression is related to tumor cell migration. It is well known that FAK phosphorylation sites (Tyr397, 407, 576, 577 861, and 925) are involved in multiple biological activities including phosphorylation of Tyr397 to promote cell motility and cell migration, Tyr576 and Tyr577 to increase catalytic activity, and Tyr925 to bind with SH2 domain of Grb2 and also to activate Ras signaling pathways [16, 39, 40]. In this study, we further investigated CUR effect on FAK in CRC tumor cell lines. We found that CUR suppresses FAK phosphorylation sites at Tyr397, 407, 576, 577, 861, and 925 in HCT-116 cells; result is partly consistent with other studies where it has been reported that CUR inhibits cell growth and migration through FAK activities [41]. CUR abrogates the chondrogenesis by downregulation of integrins and FAK leading to cytoskeleton remodeling by Akt signals [42]. CUR prevents mouse-rat hybrid retina ganglion cell migration and invasion via abrogation of FAK MMP-2, MMP-9, Rho-A, and ROCK gene expressions [29]. Cellular adhesion molecules and proteolysis activities determine the fate of tumor cell interactions with extra cellular matrix (ECM) and whether it leads to a path for tumor cell migration and angiogenesis. We also observed that CUR enhances cell adhesion ability through induction of ECM components collagens I, III, IV, and IX, laminin, and fibronectin in a concentration-dependent manner. These results suggest that CUR induces ECM components to enhance cell adhesion ability thereby preventing detachment of tumor cells. Inhibition of FAK expression leads to increase of the cell adhesion and prevention of cell migration which might be potential mechanism of antimetastasis effect of CUR. Our results suggest that CUR prevents colorectal cell progression and migration through inhibition of FAK, because downstream of FAK associates with reduced cell migration; for example, FAK deficient fibroblast shows an increased focal contact formation in culture cells [43] and reduces cell migration in glioblastoma cell lines [44]. Based on our present data, we suggest that CUR prevents CRC cell migration and invasion via inhibition of FAK activation, while enhancing ECM components to increase cell adhesion ability in CRC cell lines.

### 4.2. Curcumin Downregulates CD24 in CRC

The precise molecular mechanism of CUR on cell surface molecule CD24 has not been investigated in CRC cells. CD24 is a cell surface molecule that is employed as a gate keeper to lipid rafts within cell membrane and also promotes FAK and paxillin expression, motility, and invasion in a Src-dependent manner [17]. It was reported that CD44 is associated with FAK, paxillin, and Src activity, and the loss of CD24 expression significantly decreases cell proliferation, cell invasion, and metastasis nodules of CRC and pancreatic cancer in mice model [45]. There is possibility that inhibition of CD24 might reduce CRC cell proliferation, cell invasion, and metastasis nodules of CRC and pancreatic cancer in mice model [45]. As anticipated, our microarray analysis and qRT-PCR data indicate that CUR suppresses CD24 mRNA expression in HCT-116 cells. To our knowledge, this is the first report demonstrating that CUR has shown inhibitory effect on CD24 expression in CRC cell lines. Moreover, the CUR prevents CRC cell proliferation and metastasis through attenuation of CD24, Sp-1, and FAK activities in CRC cells, because the CD11b and CD24 associated with Sp-1 lead to promoter activity and regulation of progression of sclerosis [13, 46]. The CD24 mediates c-Src kinase for FAK phosphorylation and paxillin which in turn promote integrin-dependent adhesion and tumor cell invasion and metastasis [17]. Our results clearly implicate that CUR prevents CRC cell metastasis via inhibition of CD24 interaction with Sp-1 and FAK in CRC tumor cells.

### 4.3. Curcumin Enhances E-Cadherin Expression in CRC Cells

Cadherins regulate cytoskeleton remodeling and adhesion junctions through interaction with *β*-catenin and stabilize adhesive contact and polarization of epithelial cells. The *β*-catenin disassociates from E-cad and then translocated into nucleus from cytoplasm, where it is engaged with other transcription factors of *β*-catenin/TCF/Lef-dependent transactivation genes [22, 47]. Loss of cell-cell adhesion of tumor cells leads to invasion and metastasis into surrounding tissues [21]. It was reported that CUR could activate protein kinase D1 (PKD1) suggesting that suppressing of *β*-catenin transcriptional activity prevents growth of prostate cancer [48]. We have already reported that CUR stimulates DnaJ-like heat shock protein via activation of c-Jun NH(2)-kinase (JNK/JunD) signaling pathway and by causing upregulation of E-cad to suppress lung cancer cell invasion [27]. In this study, CUR induced E-cad expression in HCT-116 cells; results are in agreement with other studies where it was indicated that CUR attenuates nasopharyngeal carcinoma cells migration through inhibition of NF-*κ*B and by inducing the E-cad expression [49]. These results suggest that loss of E-cad adhesion leads to disruption of cell-cell adhesion and dissemination of tumor cells from epithelial cell to surrounding tissue. Therefore, CUR induced increases in E-cad expression on membrane promote the cell-cell tight junctions or intact cell-cell contacts and prevent EMT which is known to lead to inhibition of CRC cell migration, invasion, and metastasis.

### 4.4. Potential Role of EMT in Antimetastasis Effect of Curcumin

It is well known that loss of adhesion contacts in epithelial cells leads to acquired mesenchymal-like phenotype that plays central role in cancer cells invasion [50]. It was reported that EMT-related genes modulate hepatocellular carcinoma cells through CD133 expression. We have already reported that CUR induces E-cad expression through the tumor suppressor HLJ1 in lung tumor cells [27]. In this study, we found that CUR enhances the promoter activity of E-cad and thrombomodulin suggesting that an increased E-cad expression might stabilize the CRC cell-cell contact that prevents tumor cell migration. CUR suppresses the LPS induced EMT via inhibition of NF-*κ*B, snail activity, and enhanced E-cad expression in breast cancer cells [51]. It was reported that Sp-1 mediates the transforming growth factor-*β* (TNF-*β*) stimulated EMT and cell migration [52], and also downregulates E-cad and upregulation of MMP-9, thereby loss of cell-cell contact resulting in EMT, which in turn results in tumor cell invasion and metastasis. In this study, CUR inhibited CRC tumor cell metastasis via inhibition of transcription factor Sp-1 and its downstream gene expressions, while preventing cell migration through inhibition of FAK activation, CD24 expression and through increasing the ECM components and E-cad expression for enhanced cell adhesion ability in CRC cells. Suppression of transcription factor and focal adhesion molecule activities and increase in cell-cell adhesion molecule may inhibit the CRC cell metastasis. Inhibition of Sp-1 and promotion of E-cad expression may be an effective strategy for successful treatment of CUR to resistance of CRC metastasis.

### 4.5. Conclusion

Our data demonstrates the underlying molecular mechanism of CUR on CRC cell metastasis both *in vivo* and *in vitro* models. CUR is potentially inhibited by CRC cell metastasis through downregulation of Sp-1 transcription factor and its downstream signals, also prevents CRC cell invasion through suppression of FAK activation, while enhancing cell adhesion ability by increase of ECM components and promotion of E-cad expression in CRC cells. CUR is a potential novel therapeutic drug for the treatment of metastatic disease, because CUR abrogates CRC cell metastasis at different levels, particularly inhibition of transcription factor, cell adhesion molecules, and cell surface marker and enhances cell adhesion ability and cell-cell tight junctions to prevent EMT.

## Supplementary Material

Figure S1. Curcumin inhibits MMP2 and MMP9 activities.

## Figures and Tables

**Table 1:** Transcriptional regulated networks analysis of curcumin affected genes by MetaCore.

No.	Network	GO processes	Total nodes	Seed nodes	*P* value
1	Sp-1	Positive regulation of biological process (45.9%; 7.506*e* − 25), developmental process (54.1%; 2.573*e* − 24), positive regulation of cellular process (42.8%; 9.240*e* − 24), anatomical structure development (47.7%; 9.685*e* − 22), and multicellular organismal development (49.5%; 1.323*e* − 21)	223	222	0.000*E* + 00

2	HNF4-alpha	Cellular process (91.7%; 1.582*e* − 14), cellular metabolic process (66.1%; 4.500*e* − 14), cellular macromolecule metabolic process (51.1%; 7.619*e* − 12), regulation of metabolic process (49.4%; 8.376*e* − 12), and regulation of molecular function (28.3%; 3.538*e* − 11)	184	183	0.000*E* + 00

3	c-Myc	Cellular metabolic process (78.5%; 3.246*e* − 28), primary metabolic process (78.5%; 4.624*e* − 28), metabolic process (82.9%; 4.212*e* − 25), nitrogen compound metabolic process (56.9%; 8.284*e* − 23), and cellular process (96.7%; 1.088*e* − 22)	183	182	0.000*E* + 00

4	CREB1	Positive regulation of biological process (51.6%; 4.179*e* − 19), regulation of cellular process (82.8%; 4.212*e* − 18), regulation of biological process (83.6%; 1.163*e* − 16), biological regulation (85.2%; 2.132*e* − 16), and positive regulation of cellular process (45.1%; 1.518*e* − 15)	122	121	1.260*E* − 213

5	p53	Regulation of cell cycle (24.4%; 6.529*e* − 16), negative regulation of biological process (44.5%; 2.372*e* − 15), negative regulation of cellular process (42.0%; 7.092*e* − 15), anatomical structure morphogenesis (34.5%; 9.440*e* − 14), and positive regulation of biological process (45.4%; 1.493*e* − 13)	120	119	5.020*E* − 210

6	ESR1 (nuclear)	Positive regulation of biological process (51.7%; 1.418*e* − 18), positive regulation of cellular process (49.2%; 1.607*e* − 18), regulation of metabolic process (61.9%; 1.599*e* − 16), regulation of macromolecule metabolic process (54.2%; 1.174*e* − 15), and negative regulation of biological process (44.9%; 1.529*e* − 15)	118	117	1.990*E* − 206

7	AP-1	Negative regulation of biological process (55.3%; 3.643*e* − 22), negative regulation of cellular process (51.5%; 1.486*e* − 20), response to stress (53.4%; 3.747*e* − 20), positive regulation of cellular process (52.4%; 5.642*e* − 19), and positive regulation of biological process (52.4%; 5.840*e* − 17)	104	104	4.170*E* − 185

8	EGR1	Regulation of molecular function (45.5%; 4.341*e* − 19), positive regulation of biological process (54.5%; 3.071*e* − 18), developmental process (61.4%; 7.639*e* − 17), anatomical structure development (56.4%; 1.693*e* − 16), and positive regulation of cellular process (49.5%; 2.508*e* − 16)	101	100	6.280*E* − 176

9	NF-*κ*B	Positive regulation of cellular process (53.2%; 5.019*e* − 18), negative regulation of biological process (52.1%; 8.477*e* − 18), positive regulation of biological process (55.3%; 1.013*e* − 17), response to stress (52.1%; 1.554*e* − 17), and regulation of programmed cell death (36.2%; 3.551*e* − 17)	96	95	5.510*E* − 167

10	E2F1	Regulation of cell cycle (36.3%; 5.835*e* − 24), regulation of macromolecule metabolic process (69.2%; 2.822*e* − 23), regulation of primary metabolic process (70.3%; 1.619*e* − 22), regulation of cellular metabolic process (70.3%; 2.142*e* − 22), and regulation of metabolic process (73.6%; 1.740*e* − 21)	93	92	1.260*E* − 161

**Table 2:** qRT-PCR primer sequences and measurements for the potential curcumin-related genes analyzing by Affymetrix Chip.

Unigene	Symbol	Forward primer	Reverse primer	Fold change	
				Affy.	Q-PCR
Hs.116724	AKR1B10	TGGAAAAGCAACGTTCTTGGAT	TCTGGAAGTGGCTGAAATTGG	14.19	6.623
Hs.159161	ARHGDIA	GGATAAAAATCTCTTTCCGGGTTAA	CCTACCATGTAGTCAGTCTTGTCAATC	5.771	0.96
Hs.348350	DHRS1	CAAGATCCTAAGTGTGAACGTGAAGT	CAATGGAAGAGACCAGGATGACA	0.486	0.093
Hs.655387	PIK3R3	CAGACTGGAGGGAGGTGATGAT	TTGGAACTGCTGAAGTCATTGG	0.437	0.601
Hs.203717	FN1	GGAGTTGATTATACCATCACTG	TTTCTGTTTGATCTGGACCT	0.375	0.248
Hs.687708	PTK2	GCTTTGGCGGTTGCAATTAA	CACAATATGAGGATGGTCAAACTGA	2.191	0.635
Hs.116237	VAVl	TGTGAGAAGTTCGGCCTCAA	GGACAGAGCAGACAGGGTGTAGA	0.402	0.904
Hs.446336	PXN	CTGGGCAGCAACCTTTCTG	GGGCTTGAGTTGGCCTCAT	2.916	0.392
Hs.482077	ITGA2	GAAGTCTGTTGCCTGCGATGTA	GAGAGACGCCTGATTCTGAAGGT	0.446	0.331
Hs.390567	FYN	CCTTGACAACTGGAGAGACAGGTT	TTTTCGGCCAAGTTTTCCAA	0.488	0.255
Hs.531704	PRKCA	CTGCGATCACTGTGGGTCACT	GGACATTGATGACGCATTGC	0.494	0.497
Hs.433795	SHC1	AGAGTGGGCAGCCTAAGCATT	GTGGTAGCTGATAAGGTGACTGACA	2.096	0.652
Hs.521482	SHB	CAAGCTGCCCCAGGATGA	CGCTTCTCGTTGCCATTAAAC	2.081	0.588
Hs.159130	RAF1	TGTGGACAGCAGGATGATTGA	AGCCTGTTGGGCTCAGATTG	2.543	0.704
Hs.471014	TLN1	GAAGCAGAAGGGAGAGCGTAAG	GAGAACGGGCTAGCTTCACGTA	3.521	0.801
Hs.578508	ADAM10	ACTGTTCAGAACTATGGGTCTCATGT	TGTGCACTCTGTTCCAGAATCAT	0.419	0.329
Hs.20516	HDAC4	GAGGTTGAGCGTGAGCAAGAT	GTAGCGGTGGAGGGACATGT	0.245	0.167
Hs.515487	CALM1	TGGGACAGAACCCCACTGAA	CAGGAACTCCGGGAAGTCAA	2.475	0.399
Hs.275775	SEPP1	GGACTCTTTTAAATGGAAGCAAAGA	AGGAAGGAAAAAGGCAAACCA	0.242	0.215
Hs.523329	EPHB2	GGAGGGTGTCGTGACCAAGA	GACTGTGAACTGCCCATCGTTT	0.469	0.296
