# Supplementary material for: Curcumin Suppresses Metastasis via Sp-1, FAK Inhibition, and E-Cadherin Upregulation in Colorectal Cancer
Source: Evid Based Complement Alternat Med. 2013 Jul 21;2013:541695. doi: 10.1155/2013/541695 (PMC3736531; doi:10.1155/2013/541695)
Supplement: Supplementary file 1 — Figure S1. Curcumin inhibits MMP2 and MMP9 activities. [file 541695.f1.doc]

**Supplementary Method**

**Zymography assay**

1 x 10^6^ HCT-116 cells were seeded in six-well plates and incubated for 24 h at 37°C to 80% confluence. Cells were washed twice with PBS buffer and cultured for an additional 24 h in serum-free medium followed by collecting the supernatant. The activities of MMP2 and MMP9 in the resulting supernatants were analyzed by 8% SDS-PAGE with 0.5 mg/ml gelatin. The gel was washed twice for 30 mins at room temperature in washing buffer, incubated for 24h at 37°C, and stained with 0.1% Coomassie Brilliant Blue R-250.

**Supplementary Data S1**


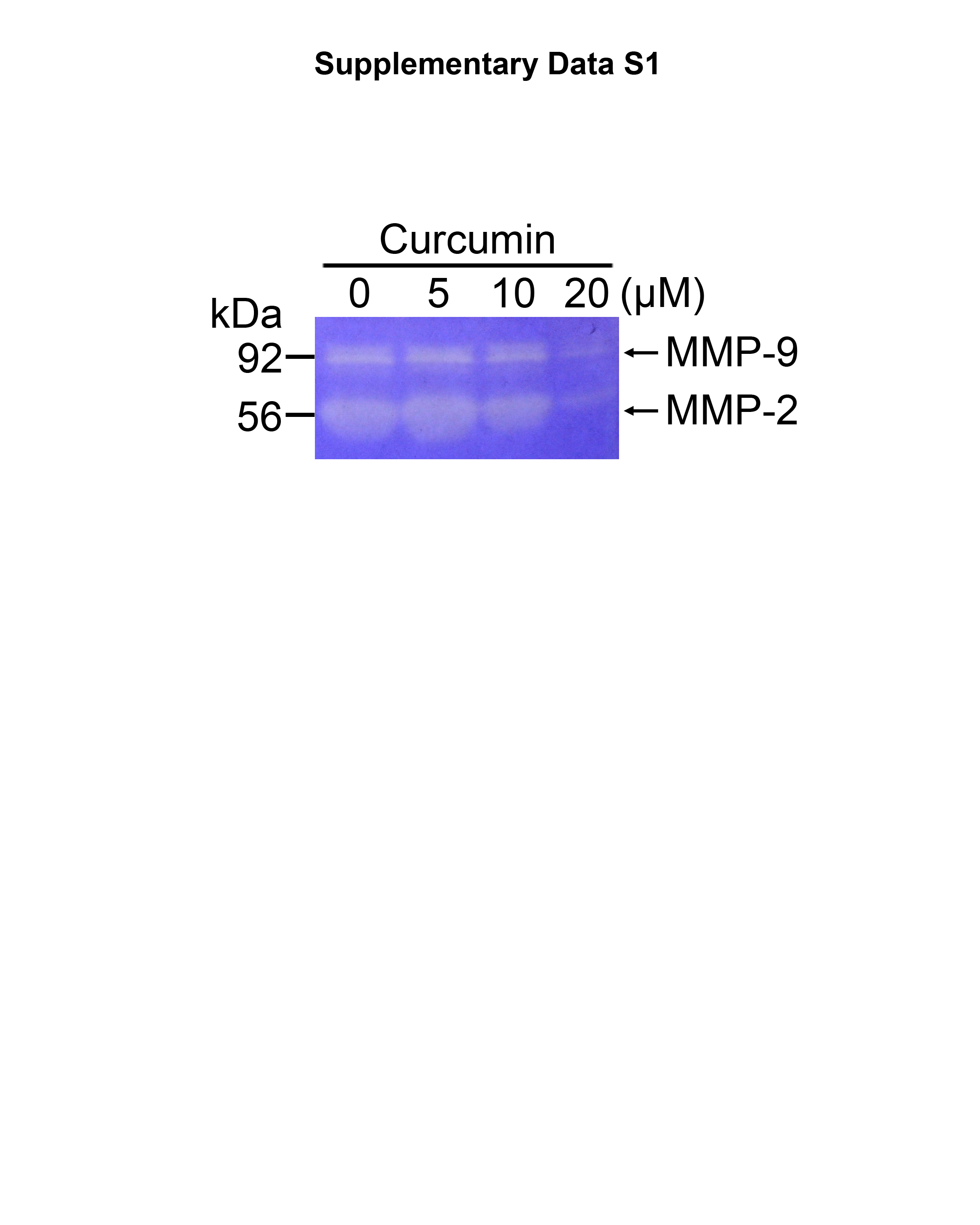


**Figure S1. Curcumin inhibits MMP2 and MMP9 activities.**

MMP2 and MMP9 activities were inhibited in curcumin-treated HCT-116 cells determined by zymography assay. The intensity of blank zone in gel represents the activity of MMPs.
